# Supplementary material for: PP6 phosphatase and Elongator contribute to kinesin 5-dependent spindle assembly by controlling microtubule regulators levels
Source: PLoS Genet. 2025 Oct 7;21(10):e1011596. doi: 10.1371/journal.pgen.1011596 (PMC12520374; doi:10.1371/journal.pgen.1011596)
Supplement: S1 Table — (DOCX) [file pgen.1011596.s001.docx]

**SUPPLEMENTARY INFORMATION**

**Table S1. Table of strains used in this study.**

| **Strain** | **Genotype** | **Source** |
| --- | --- | --- |
| SR1.01 | *h*- wild type *leu1-32 ura4-D18* | Lab stock |
| SR1.25 | *h*+ *ppe1*∆*::kanMX6 leu1-32 ura4-D18 ade6-* | This study |
| SR1.32 | *h*- *cut7-24:natMX6 leu1-32 ura4-D18* | Lab stock |
| SR1.63 | *h*+ *cut7-24:natMX6 ppe1*∆*::kanMX6 leu1-32 ura4-D18 ade6-* | This study |
| SR1.31 | *h*- *cut7-22:natMX6 leu1-32 ura4-D18 ade6-* | This study |
| SR20.43 | *h-* *cut7-22:natMX6 ppe1*∆*::kanMX6 leu1-32 ura4-D18 ade6-* | This study |
| SR6.13 | *h*- *sid4-mCherry:hphMX6 leu1-32 ura4-D18* | Lab stock |
| SR5.71 | *h*- *sid4-mCherry:hphMX6 ppe1*∆*::kanMX6 leu1-32 ura4-D18 ade6-* | This study |
| SR21.21 | *h+* *sid4-mCherry:hphMX6 cut7-22:natMX6 leu1-32 ura4-D18 ade6-* | This study |
| SR21.18 | *h+* *sid4-mCherry:hphMX6 cut7-22:natMX6 ppe1*∆*::kanMX6 leu1-32 ura4-D18 ade6-* | This study |
| SR1.09 | *h-* *cut7-Envy:kanMX6 mCherry-atb2:hphMX6 leu1-32 ura4-D18* | This study |
| SR35.07 | *h-* *cut7-Envy:kanMX6 mCherry-atb2:hphMX6 ppe1*∆*::natMX6 leu1-32 ura4-D18 ade6-* | This study |
| SR31.30 | *h+* *pkl1-neonGreen:kanMX6 mCherry-atb2:hphMX6 leu1-32 ura4-D18* | This study |
| SR31.34 | *h+* *pkl1-neonGreen:kanMX6 mCherry-atb2:hphMX6 ppe1*∆*::natMX6 leu1-32 ura4-D18* | This study |
| SR20.63 | *h+ elp4*∆*::kanMX6 leu1-32 ura4-D18* | This study |
| SR21.24 | *h- cut7-22:natMX6 elp4*∆*::kanMX6 leu1-32 ura4-D18 ade6-* | This study |
| SR21.36 | *h+* *sid4-mCherry:hphMX6 elp4*∆*::kanMX6 leu1-32 ura4-D18 ade6-* | This study |
| SR21.44 | *h+* *sid4-mCherry:hphMX6 cut7-22:natMX6 elp4*∆*::kanMX6 leu1-32 ura4-D18 ade6-* | This study |
| SR20.49 | *h*+ *elp3*∆*::natMX6 leu1-32* | This study |
| SR25.26 | *h*+ *elp3-Y527A Y528A::hphMX6* | This study |
| SR2.12 | *h*+ *ppe1*∆*::natMX6 leu1-32 ura4-D18 ade6-* | This study |
| SR28.19 | *h*+ *mph1*∆*::kanMX6 leu1-32 ura4-D18 ade6-* | This study |
| SR28.27 | *h*+ *mph1*∆*::kanMX6 elp3::natMX6 leu1-32 ura4-D18 ade6-* | This study |
| SR28.29 | *h-* *mph1*∆*::kanMX6 elp3-Y527A Y528A::hphMX6 leu1-32 ura4-D18* | This study |
| SR28.23 | *h*+ *mph1*∆*::kanMX6 ppe1*∆*::natMX6 leu1-32 ura4-D18 ade6-* | This study |
| SR31.80 | *h-* *klp2-neonGreen:kanMX6 mCherry-atb2:hphMX6 leu1-32 ura4-D18* | This study |
| SR35.24 | *h-* *klp2-neonGreen:kanMX6 mCherry-atb2:hphMX6 ppe1*∆*::natMX6 leu1-32 ura4-D18* | This study |
| SR35.26 | *h-* *klp2-neonGreen:kanMX6 mCherry-atb2:hphMX6 elp3*∆*::natMX6 leu1-32 ura4-D18* | This study |
| SR30.29 | *h-* *klp2-13xMyc:hphMX6 leu1-32 ura4-D18 ade6-* | This study |
| SR30.77 | *h-* *klp2-13xMyc:hphMX6 ppe1*∆*::natMX6 leu1-32 ura4-D18 ade6-* | This study |
| SR38.44 | *h+* *klp2-13xMyc:hphMX6 elp3*∆*::natMX6 leu1-32 ura4-D18 ade6-* | This study |
| SR29.69 | *h-* *alp7-neonGreen:kanMX6 mCherry-atb2:hphMX6 leu1-32 ura4-D18* | This study |
| SR29.71 | *h-* *alp7-neonGreen:kanMX6 mCherry-atb2:hphMX6 ppe1*∆*::natMX6 leu1-32 ura4-D18* | This study |
| SR35.34 | *h-* *alp7-neonGreen:kanMX6 mCherry-atb2:hphMX6 elp3*∆*::natMX6 leu1-32 ura4-D18* | This study |
| SR9.22 | *h+* *alp7-3xGFP:kanMX6 mCherry-atb2:hphMX6 leu1-32 ura4-D18 ade6-* | This study |
| SR13.77 | *h-* *alp7-3xGFP:kanMX6 mCherry-atb2:hphMX6 ppe1*∆*::natMX6 leu1-32 ura4-D18 ade6-* | This study |
| SR20.12 | *h+* *alp7-3xGFP:kanMX6 mCherry-atb2:hphMX6 elp3*∆*::natMX6 ura4-D18 ade6-* | This study |
| SR1.12 | *h-* *ase1-GFP:kanMX6 mCherry-atb2:hphMX6 leu1-32 ura4-D18 ade6-* | This study |
| SR29.74 | *h-* *ase1-GFP:kanMX6 mCherry-atb2:hphMX6 ppe1*∆*::natMX6 leu1-32 ura4-D18 ade6-* | This study |
| SR32.14 | *h-* *ase1-GFP:kanMX6 mCherry-atb2:hphMX6 elp3*∆*::natMX6 leu1-32 ura4-D18 ade6-* | This study |
| SR54.03 | *h-* *ase1-13xMyc:hphMX6 leu1-32 ura4-D18 ade6-* | This study |
| SR54.10 | *h-* *ase1-13xMyc:hphMX6 ppe1*∆*::natMX6 leu1-32 ura4-D18 ade6-* | This study |
| SR54.12 | *h+* *ase1-13xMyc:hphMX6 elp3*∆*::natMX6 ade6-* | This study |
| SR25.30 | *h+* *elp4-S114A::kanMX6 leu1-32 ura4-D18 ade6-* | This study |
| SR29.76 | *h+* *ppe1*∆*::natMX6 elp4-S114A::kanMX6 leu1-32 ura4-D18 ade6-* | This study |
| SR25.10 | *h*- *cut7-22:hphMX6 leu1-32 ura4-D18 ade6-* | This study |
| SR25.43 | *h+* *cut7-22:hphMX6 ppe1*∆*::natMX6 leu1-32 ura4-D18 ade6-* | This study |
| SR26.59 | *h-* *cut7-22:hphMX6 elp4-S114A::kanMX6 leu1-32 ura4-D18 ade6-* | This study |
| SR26.61 | *h-* *cut7-22:hphMX6 ppe1*∆*::natMX6 elp4-S114A::kanMX6 leu1-32 ura4-D18 ade6-* | This study |
| SR35.30 | *h-* *klp2-neonGreen:kanMX6 mCherry-atb2:hphMX6 elp4-S114A::kanMX6 leu1-32 ura4-D18* | This study |
| SR35.32 | *h-* *klp2-neonGreen:kanMX6 mCherry-atb2:hphMX6 ppe1*∆*::natMX6 elp4-S114A::kanMX6 leu1-32 ura4-D18* | This study |
| SR35.58 | *h-* *alp7-neonGreen:kanMX6 mCherry-atb2:hphMX6 elp4-S114A::kanMX6 leu1-32 ura4-D18 ade6-* | This study |
| SR35.60 | *h-* *alp7-neonGreen:kanMX6 mCherry-atb2:hphMX6 ppe1*∆*::natMX6 elp4-S114A::kanMX6 leu1-32 ura4-D18 ade6-* | This study |
| SR54.29 | *h-* *ase1-GFP:kanMX6 mCherry-atb2:hphMX6 elp4-S114A::kanMX6 leu1-32 ura4-D18 ade6-* | This study |
| SR54.31 | *h-* *ase1-GFP:kanMX6 mCherry-atb2:hphMX6 ppe1*∆*::natMX6 elp4-S114A::kanMX6 leu1-32 ura4-D18 ade6-* | This study |
| **Supplementary Figures** | | |
| SR1.01 | *h*- wild type *leu1-32 ura4-D18* | Lab stock |
| SR1.28 | *h*+ *ekc1*∆*::kanMX6* | This study |
| SR1.32 | *h*- *cut7-24:natMX6 leu1-32 ura4-D18* | Lab stock |
| SR1.64 | *h*+ *cut7-24:natMX6 ekc1*∆*::kanMX6 ade6-* | This study |
| SR20.10 | *h+* *cut7-3xHA:hphMX6 leu1-32 ura4-D18 ade6-* | This study |
| SR25.16 | *h+* *cut7-3xHA:hphMX6 ppe1*∆*::kanMX6 leu1-32 ura4-D18 ade6-* | This study |
| SR20.59 | *h+* *cut7-3xHA:hphMX6 nda3-KM311 leu1-32 ura4-D18* | This study |
| SR20.60 | *h+* *cut7-3xHA:hphMX6 ppe1*∆*::kanMX6 nda3-KM311 leu1-32 ura4-D18 ade6-* | This study |
| SR30.53 | *h+* *pkl1-13xMyc:hphMX6 leu1-32 ura4-D18 ade6-* | This study |
| SR32.28 | *h-* *pkl1-13xMyc:hphMX6 ppe1*∆*::natMX6 leu1-32 ura4-D18 ade6-* | This study |
| SR38.34 | *h+* *pkl1-13xMyc:hphMX6 nda3-KM311 leu1-32 ura4-D18* | This study |
| SR38.36 | *h+* *pkl1-13xMyc:hphMX6 ppe1*∆*::natMX6 nda3-KM311 leu1-32 ura4-D18 ade6-* | This study |
| SR14.09 | *h*- *cut7-24:kanMX6 leu1-32 ura4-D18* | This study |
| SR19.74 | *h+* *cut7-24:kanMX6 elp3::natMX6 leu1-32 ade6-* | This study |
| SR20.46 | *h-* *cut7-22:natMX6 elp3::kanMX6 ade6-* | This study |
| SR2.56 | *h+* *GFP-atb2:hphMX6 leu1-32 ura4-D18 ade6-* | Lab stock |
| SR38.38 | *h-* *GFP-atb2:hphMX6 ppe1*∆*::natMX6 leu1-32 ura4-D18 ade6-* | This study |
| SR38.40 | *h-* *GFP-atb2:hphMX6 elp3*∆*::natMX6 leu1-32 ura4-D18 ade6-* | This study |
| SR38.42 | *h-* *GFP-atb2:hphMX6 elp3-Y527A Y528A::hphMX6 leu1-32 ura4-D18 ade6-* | This study |
| SR23.16 | *h+* *elp3-GFP:natMX6 mCherry-atb2:hphMX6 leu1-32 ura4-D18* | This study |
| SR22.71 | *h+* *elp4-GFP:natMX6 mCherry-atb2:hphMX6 leu1-32 ura4-D18* | This study |
| SR6.03 | *h+* *ppe1*∆*::hphMX6 leu1-32 ura4-D18 ade6-* | This study |
| SR2.40 | *h-* *klp2*∆*::kanMX6 leu1-32 ura4-D18 ade6-* | This study |
| SR8.31 | *h+* *ppe1*∆*::hphMX6 klp2*∆*::kanMX6 leu1-32 ura4-D18 ade6-* | This study |
| SR28.04 | *h+* *cut7-22:hphMX6 klp2*∆*::kanMX6 leu1-32 ura4-D18 ade6-* | This study |
| SR28.13 | *h+* *cut7-22:hphMX6 ppe1*∆*::natMX6 klp2*∆*::kanMX6 leu1-32 ura4-D18 ade6-* | This study |
| SR2.28 | *h+* *alp7*∆*::ura4+ leu1-32 ura4-D18 ade6-* | Lab stock |
| SR2.34 | *h+* *ppe1*∆*::natMX6 alp7*∆*::ura4^+^ leu1-32 ura4-D18* | This study |
| SR28.08 | *h+* *cut7-22:hphMX6 alp7*∆*::ura4^+^ leu1-32 ura4-D18* | This study |
| SR29.56 | *h+* *cut7-22:hphMX6 ppe1*∆*::kanMX6 alp7*∆*::ura4^+^ leu1-32 ura4-D18* | This study |
| SR2.30 | *h*+ *ase1*∆:*:kanMX6 leu1-32 ura4-D18* | This study |
| SR2.35 | *h*+ *ase1*∆:*:kanMX6 ppe1*∆*::natMX6 leu1-32 ura4-D18 ade6-* | This study |
| SR31.52 | *h-* *cut7-22:hphMX6 ase1*∆:*:kanMX6 leu1-32 ura4-D18 ade6-* | This study |
| SR54.33 | *h-* *cut7-22:hphMX6 ppe1*∆*::natMX6 ase1*∆:*:kanMX6 leu1-32 ura4-D18 ade6-* | This study |
| PPG1.01 | *h*- wild type (972) | Lab stock |
| SR40.01 | *h*+ *ppe1*∆*::natMX6* | This study |
| SR40.12 | *h*+ *elp3*∆*::natMX6* | This study |
| SR40.03 | *h*+ *elp4-S114A:kanMX6* | This study |
| SR40.05 | *h*+ *ppe1*∆*::natMX6 elp4-S114A:kanMX6* | This study |
