# Supplementary figures and images for: PP6 phosphatase and Elongator contribute to kinesin 5-dependent spindle assembly by controlling microtubule regulators levels
Source: PLoS Genet. 2025 Oct 7;21(10):e1011596. doi: 10.1371/journal.pgen.1011596 (PMC12520374; doi:10.1371/journal.pgen.1011596)

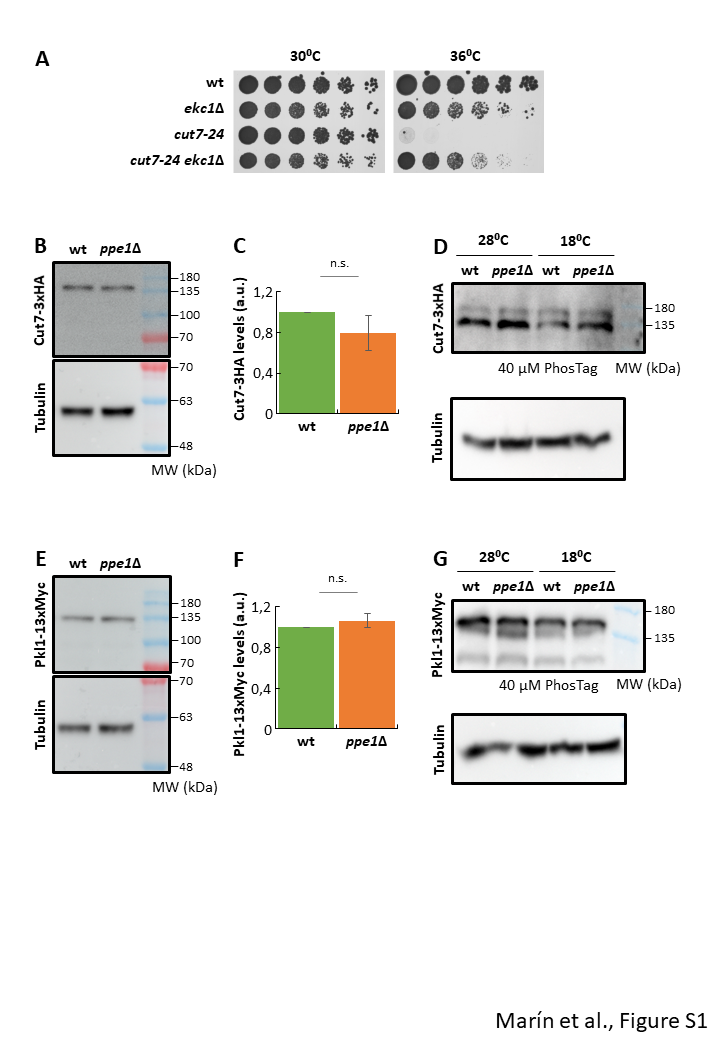

Supplement: S1 Fig — A: Serial dilution assay of the wild type, ekc1∆, cut7–24 and cut7–24 ekc1∆ strains incubated for 3 days at the indicated temperatures. B: SDS PAGE analysis of Cut7-3xHA levels on wild type or ppe1∆ cells. The upper panel corresponds to the blot incubated with an anti-HA antibody, while the bottom panel corresponds to the blot incubated with an anti-tubulin antibody. C: Plot showing the total Cut7-3xHA levels relative to the corresponding tubulin levels in wild type and ppe1∆ cells. n for wild type: 3; n for ppe1∆: 3. a.u.: arbitrary units. Statistical significance was determined using a T-test (p = 0.086). D: Upper panel, PhosTag SDS PAGE analysis of Cut7-3xHA in asynchronous and mitotically blocked nda3-KM311 cells in the presence or absence of Ppe1. Bottom panel, the same samples submitted to SDS PAGE analysis using an anti-tubulin antibody. E: SDS PAGE analysis of Pkl1-13xMyc levels on wild type or ppe1∆ cells. The upper panel corresponds to the blot incubated with an anti-Myc antibody, while the bottom panel corresponds to the blot incubated with an anti-tubulin antibody. F: Plot showing the total Pkl1-13xMyc levels relative to the corresponding tubulin levels in wild type and ppe1∆ cells. n for wild type: 3; n for ppe1∆: 3. a.u.: arbitrary units. Statistical significance was determined using a T-test (p = 0.121). G: Upper panel, PhosTag SDS PAGE analysis of Pkl1-13xMyc in asynchronous and mitotically blocked nda3-KM311 cells in the presence or absence of Ppe1. Bottom panel, the same samples submitted to SDS PAGE analysis using an anti-tubulin antibody. (TIF) [file pgen.1011596.s003.TIF]

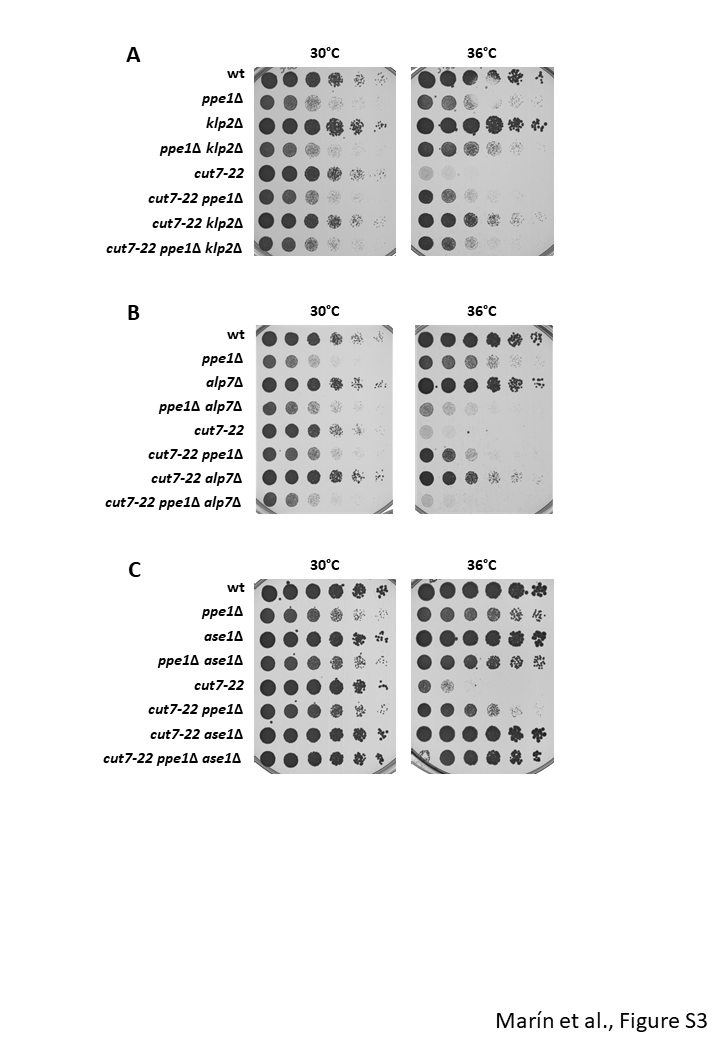

Supplement: S3 Fig — A: Serial dilution assay of the wild type, ppe1∆, klp2∆, ppe1∆ klp2∆, cut7–22, cut7–22 ppe1∆, cut7–22 klp2∆ and cut7–22 ppe1∆ klp2∆ strains incubated for 3 days at the indicated temperatures. B: Serial dilution assay of the wild type, ppe1∆, alp7∆, ppe1∆ alp7∆, cut7–22, cut7–22 ppe1∆, cut7–22 alp7∆ and cut7–22 ppe1∆ alp7∆ strains incubated for 3 days at the indicated temperatures. C: Serial dilution assay of the wild type, ppe1∆, ase1∆, ppe1∆ ase1∆, cut7–22, cut7–22 ppe1∆, cut7–22 ase1∆ and cut7–22 ppe1∆ ase1∆ strains incubated for 3 days at the indicated temperatures. (TIF) [file pgen.1011596.s005.TIF]

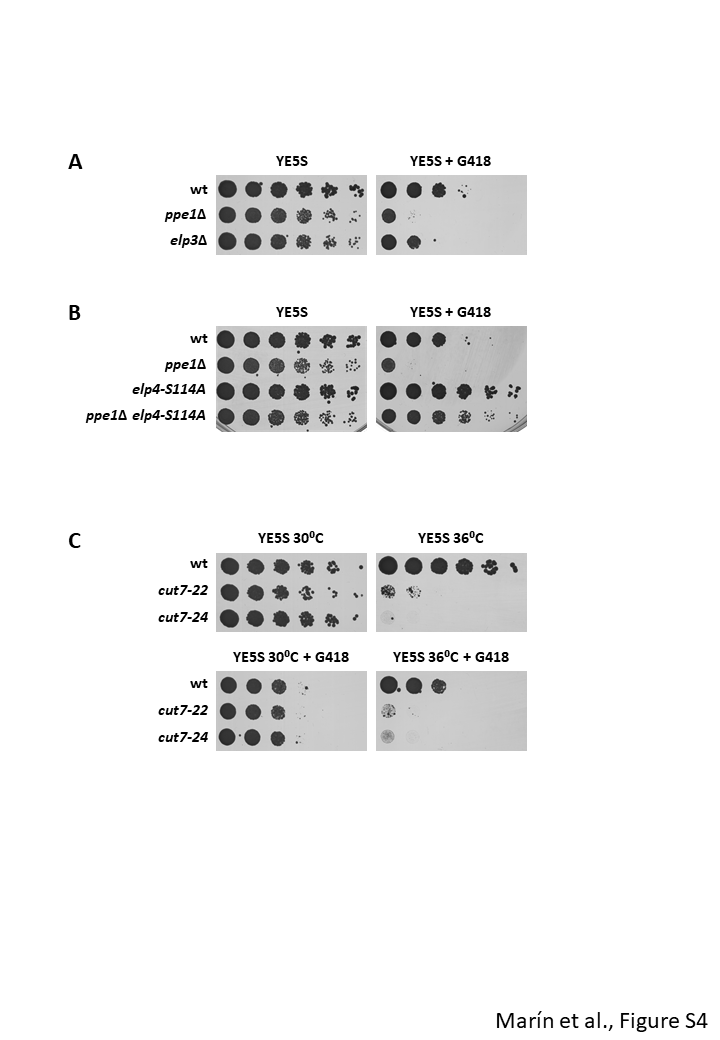

Supplement: S4 Fig — A: Serial dilution assay of the wild type, ppe1∆ and elp3∆ strains incubated for 3 days in YE5S of YE5S plates supplemented with 5 μg/ml of G418 at 30 °C. B: Serial dilution assay of the wild type, ppe1∆, elp4-S114A and ppe1∆ elp4-S114A strains incubated for 3 days in YE5S of YE5S plates supplemented with 5 μg/ml of G418 at 30 °C. C: Serial dilution assay of the wild type, cut7–22 and cut7–24 strains incubated for 3 days at the indicated temperatures supplemented with 5 μg/ml of G418. (TIF) [file pgen.1011596.s006.TIF]
