# Supplementary material for: PP6 phosphatase and Elongator contribute to kinesin 5-dependent spindle assembly by controlling microtubule regulators levels
Source: PLoS Genet. 2025 Oct 7;21(10):e1011596. doi: 10.1371/journal.pgen.1011596 (PMC12520374; doi:10.1371/journal.pgen.1011596)
Supplement: Attachment — Submitted filename: Rebuttal Letter.pdf [file pgen.1011596.s008.pdf]

Sergio Rincon
IBFG
CSIC / University of Salamanca
37007, Salamanca, Spain

Salamanca, July 31, 2025

Manuscript Number: PGENETICS-D-25-00113
MS TITLE: "PP6 phosphatase and Elongator contribute to kinesin 5-dependent spindle assembly by controlling microtubule regulators levels"

Dear Ms. Szabo and the PLOS Genetics Editorial Board,

Please find attached our revised manuscript. We think we have now addressed most of the reviewers' comments.

In our opinion, the major concern of the reviewers was figure presentation and the interpretation of specific results. To solve this, we have reorganized the figure layout, repeated western blots in a more quantitative manner and included new statistical quantification.

First, we have taken part of supplementary figures 1 and 3 into the main figures 1 and 3, respectively, to backup the manuscript text without the need of having the supplementary figures on-hand. We agree that, even though they are negative results, these data are extensively mentioned in the main text.

Second, we have added statistics comparing the accumulation of the proteins analyzed in this work, to clarify the significance of our results.

Third, all the western blots performed to analyze the levels of the proteins of interest have been repeated using a chemiluminescence imaging system, to get more quantitative data.

Finally, we strongly acknowledge the third reviewer comment on our interpretation of the *ase1*ΔC mutant. It was previously stated that this truncated version of the Ase1 microtubule bundler showed a decreased accumulation of Cls1 to the mitotic spindle (Bratman et al., Dev Cell, 2007). Indeed, this observation has been put into question in a recent paper, which shows how early Cls1 recruitment to the spindle is Ase1-independent (Ebina et al., Biol Open, 2019). This prompted us to find another way to check if the reduction in Cls1 levels facilitates spindle bipolarity in a *cut7-22* mutant. To do so, we have created an auxin degron version of Cls1. Our results show that reduced

levels of Cls1 does not rescue *cut7-22*, suggesting that Cls1 contribution to spindle bipolarity is especially critical when Cut7 function is compromised (rebuttal letter Figure 1). On the contrary, we have found that the complete absence of Ase1 fully suppresses *cut7-22* thermo-sensitivity. Interestingly, we have analyzed Ase1 levels in the absence of PP6 and Elongator activity, and found a moderate decrease, which, together with the reduction in Klp2 and Alp7, explains the suppression of *cut7-22* thermo-sensitivity when combined with *ppe1*Δ or *elp3*Δ mutants. Therefore, the data related to Cls1 has now been replaced for that of Ase1, given that does not explain the suppression of this phenotype.

Additionally, we have addressed all minor points raised by the reviewers.

A detailed reply to the reviewers' concerns and questions is provided below.

We hope that our manuscript is now suitable for publication in PLOS Genetics.

Best regards,

Sergio Rincon

Specific answers to reviewers' comments

Reviewer #1

Major points:

1. **I felt that the majority of the first three supplementary figures should be included in the main text. Significant sections of text refer to data in these figures, which is important for the progression of the experiments described. Since, there are currently only four main figures, I would recommend moving these supplementary figures into the main text. For example, the majority of the text from lines 321-334 is discussing data in Figure S3. I don't think readers should have to delve into supplementary data, for figures that so strongly support the text. I am almost tempted to suggest inclusion of the screen results as a table in the main text – it is not huge and contains interesting other mutations e.g. Polo kinase (Plo1).**

We thank the reviewer for this comment. A significant part of the experimental work had been included into supplementary figures, mainly because they were negative results. However, we do agree that the text reads easier with the data on-hand, without the need of having the supplementary data. On that sense, we have transferred previous figures S1A, S1D, S3A, S3C and new Ase1 data into figures 1 and 3, respectively. New figure 1 feels now too packed to include the screen results; however, we have included some relevant results of the screen in the main text, (current lines 167-168).

2. Figure 1C it should be possible to calculate a statistic (Fishers exact test?) to assure readers that the difference discussed in lines 176-177 is robust based on the numbers. It would be good to include the n numbers on the figure. This also applies to Figure 2C.

We agree with the reviewer that data shown in figures 1C and 2C needed statistical analysis to strengthen our conclusions. To do so, we have repeated twice our experiments to have three biological replica, which has significantly increased the number of cells analyzed. We have now shown the average percentage of spindle bipolarity in each condition, and performed a T-test to confirm that the absence of Ppe1 significantly rescues *cut7-22* defect in spindle bipolarity at restrictive temperature. To avoid overloading figures with numbers, we have kept the n numbers and the p values in the figure legends.

3. Figure 1D I assume that cut7-22 is not included in this figure, since mostly these cells do not form bipolar spindles (as per Figure 1C) – this should be mentioned.

We have included a sentence clarifying this in current lines 185-187 ("*cut7-22* cells were not included in this analysis, given that most of the cells produced a monopolar spindle and eventually lysed upon constriction of the cytokinetic ring on the dividing nucleus.").

4. The rescue to temperature tolerance of cut7-22 cells by elp4Δ is modest at best (but evident) – I would replace "partially" line 238, with "to a small extent". The rescue for elp3Δ shown in the supplementary data is much clearer.

We agree with the reviewer that the suppression of *cut7-22* thermosensitive by the absence of Elongator activity is limited, but consistent. The suggestion of the reviewer has been included in the current line 249.

5. Figure 2D. I am struggling to see the synergy of the mutants. mph1Δ mutants are quite sensitive to MBC (especially at 3 µg/ml). When combined with elp3Δ, elp3-YY527AA or ppe1Δ, I am not convinced they are more sensitive than mph1Δ alone (note the double mutants grow poorly even on DMSO). Is there some way to quantify these more carefully?

We thank the reviewer for the comment. Indeed, *mph1*Δ *ppe1*Δ cells grow slower than the single mutants, which makes difficult to see the effect of MBC on the strains' growth. We have repeated the serial dilution, changing the strains' order, leaving an additional day of incubation and showing the 2 µg/ml concentration. This makes the differences more evident now, reinforcing the idea that Ppe1 may have additional roles than Elongator in mitosis (related to protein translation or not).

6. I don't see any statistical analysis to back up the conclusion that Alp7 intensity is reduced by 25% Figure 3B & line 310/311. I realise this reduction is not likely relevant for the cut7 mutant by PP6 mutations.

We agree with the reviewer that the reduction of Alp7 levels in *ppe1*Δ cells is modest in our microscopy analysis. We have now included statistical analysis on all our protein accumulation data. In particular, we have performed a repeated measures ANOVA that shows a statistical difference in the protein accumulation at the mitotic spindle in *ppe1*Δ cells compared to wild type cells. However, this analysis did not find statistical differences in *elp3*Δ cells compared to wild type cells. Moreover, Elongator hyperactivation cannot compensate for the loss of Ppe1 (now included in current figure 4C). Altogether, this may explain why the absence of Ppe1 suppresses *cut7* mutant thermo-sensitivity to a higher extent than the lack of Elp3.

7. Figure 4B and 4C – do the statistical test back up that elp4-S114A "weakly increased" (line 365) the levels of Klp2 and Cls1 in a ppe1Δ mutant? I think it is "n.s." for Klp2 and I don't see a statistical test in Figure 4C.

We apologize for the lack of statistical analysis in Figure 4C. As discussed before, we have done a repeated measures ANOVA to test if the differences detected were significant or not. As explained above, given that the reduction in Cls1 levels do not account for the suppression of *cut7-22* thermo-sensitivity, and that the absence of Ase1 does allow *cut7-22* growth at restrictive temperature, we have analyzed Ase1 accumulation in *ppe1*Δ cells. Our results show

a modest but significant reduction of Ase1 levels in *ppe1*Δ mutant, which is not significantly suppressed by combination with the Elp4 hyperactive mutant, Elp4-S114A (figure 4ID). A similar result was found for Alp7. Therefore, our conclusion reads now “Our analysis showed that Klp2, Alp7 or Ase1 levels in cells lacking Ppe1 were not significantly increased by the expression of the hyperactive *elp4-S114A* allele” in lines 383-385.

Minor points:

- ***Line 39 “recently involved in” to “recently been implicated in”***

This has been fixed.

- ***Line 153 fix “unable of assembling”***

This has been replaced by “cannot assemble”.

- ***Line 227 “consequence” to “a consequence”***

This has been fixed.

- ***The strains list needs fixing; lots of highlighted text and a row labelled “supplementary figures”***

We apologize for this mistake. This has been solved.

Reviewer #2

Major points:

1. Too much background information was presented in the abstract. Perhaps, more results and conclusive statements, instead of background information, should be provided.

We thank the reviewer for this comment. In fact, we think it is important to properly set the scenario of this work, which consists of the mechanisms of bipolar spindle assembly. Nevertheless, we agree a more detailed information of the results may gather a broader readership. In that sense, we have now specified the microtubule regulators whose levels are affected in the absence of PP6 or Elongator activity, contributing to bipolar spindle formation when kinesin 5 activity is compromised.

2. It is unclear how the absence of Ppe1 influences the protein level of Klp2.

Ppe1 acts as a positive regulator of the Elongator complex (Candiracci et al., Sci Adv, 2019). Elongator complex modifies uridine 34 on tRNAs so that the codon – anticodon interaction is robust enough even when the wobble position of the codon is an adenine (AAA, GAA or CAA). These codons, which code for lysine, glutamate and glutamine, respectively, are synonyms of AAG, GAG and CAG, respectively. In the absence of Elongator (or Ppe1), translation of mRNAs with high use of AAA (and also GAA, but not CAA, according to Bauer et al., Cell Rep, 2012) is compromised. This is the case for *klp2* mRNA, which contains an AAA/AAG codon usage for lysine of 0.66/0.34, and a GAA/GAG codon usage for glutamate of 0.74/0.26. Therefore, the translation of this mRNA becomes highly dependent of the Elongator complex and Ppe1. To make the role of Ppe1 and Elongator on protein translation clearer, we have added a paragraph in the introduction (lines 132-137), which reinforces the statement of lines 311-313 that explains the reduction in Klp2 levels detected in *ppe1*Δ and *elp3*Δ mutants.

3. It is unclear why the absence of either Alp7 or Ppe1 suppresses the effects of cut7-22 but double-deletion alp7 and ppe1 cannot.

As stated in lines 330-331, *ppe1*Δ shows a very strong genetic interaction with *alp7*Δ. In the previous version of our paper, we showed that *ppe1*Δ cells have a mild reduction in Cls1 levels. Indeed, we have found that *alp7*Δ is also synthetic to the *cls1* thermo-sensitive mutant *cls1-36* (Figure RB1).

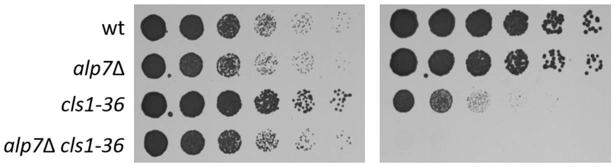

Figure RB1. *alp7*Δ is strongly synthetic to the *cls1-36*

In our new version of the paper, we have replaced Cls1 data for that of Ase1, since a reduction in Cls1 levels do not seem to explain *cut7-22* themo-sensitivity suppression. The absence of Ase1, in contrast, rescues *cut7-22* viability at restrictive temperature, and the levels of this protein are also reduced in the absence of Ppe1 or Elongator. It turns out that the double deletion *alp7*Δ *ase1*Δ is lethal (Thadani et al., 2009). In summary, it is possible that the strong genetic interaction shown by *alp7*Δ and *ppe1*Δ may stem from the reduced levels of both Cls1 and Ase1 in the absence of Ppe1. To highlight this possibility, we have added this sentence "This genetic interaction may be the consequence of reduced levels of specific proteins in the absence of Ppe1, which might be deleterious in the absence of Alp7" in lines 332-334.

4. Does the absence of Ppe1 influences the function of the elongator complex in fission yeast?

As stated in lines 230-231, Candiracci and collaborators (2019, Sci Adv) have proposed that Ppe1 activates the Elongator complex by counteracting the inhibiting phosphorylation of the kinase Gsk3 on the residue S144 of Elp4. In this work, it was shown that tRNA modifications were strongly reduced in *ppe1*Δ cells, to a similar extent to the *elp4*Δ mutant. Moreover, they demonstrate that hyperactivation of Elongator (by using the *elp4-S114A* mutant), partially rescues *ppe1*Δ phenotypes (rapamycin sensitivity, in this case).

5. How do the authors explain the additive effects of the double deletion-mutants mph1-ppe1-deletion and mph1-elp3-deletion?

Mph1 is the central kinase of the Spindle Assembly Checkpoint, a pathway that halts mitotic progression upon mitotic defects, such as chromosome capture failure. The absence of Mph1 *per se* is not deleterious in fission yeast; however, compromising mitotic progression by using microtubule depolymerizing drugs, such as MBC, has a strong effect on *mph1*Δ cells. This provides a tool to uncover genetic interactions that would be otherwise undetectable. Our interpretation of the additive effects of *mph1*Δ to *elp3* mutants, and especially to *ppe1*Δ is that both Ppe1 and Elp3 have a role in the control of mitotic progression. Our data shows that the lack of PP6 or Elongator activity results in reduced levels of microtubule regulators, which, in turn, affect to some extent the fidelity of mitosis. To reinforce our interpretation of the result, we have added the following statement in lines 279-280: "This result points towards a role of Ppe1 and Elongator in the control of mitosis progression".

Reviewer #3

Major points:

1. My major concern is the presentation and interpretation of gel and blot results.

1a. The authors show subsets with only specific bands of the gels or blots, and the molecular weight is indicated only by labels on the bands with no markers shown. The authors should show the full gels and blots for their results with the markers shown for a more rigorous presentation. This will allow the reader to see, for example, whether degradation products are present and how well their antibodies work.

We thank the reviewer for the comment. Indeed, problems in protein translation may result in degradation of not properly folded proteins. However, in the conditions we obtained our samples, we did not detect degradation products at a significant level. Nevertheless, we have increased the width of our blots, following the reviewer recommendations. Similarly, we have included the molecular weight marker bands. Full blots are shown in figure RB2.

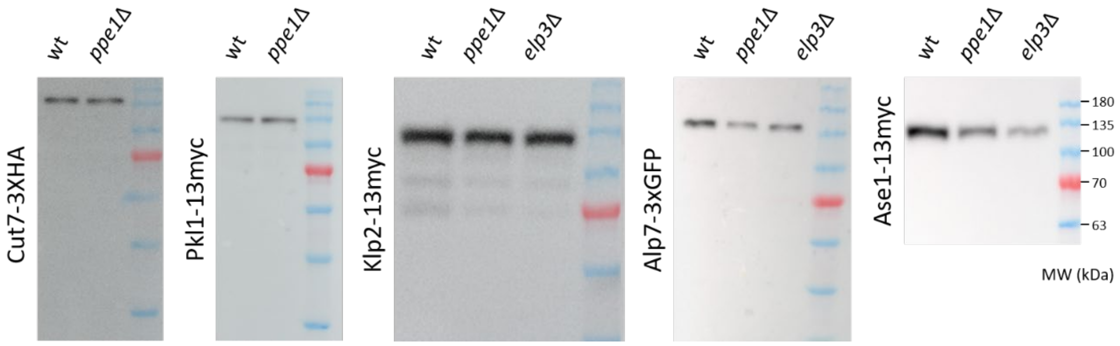

Figure RB2. Protein degradation was not typically observed in our blots.

1b. Further, the authors do not present total protein level quantifications for their Western blots. These need quantification: volume-to-volume comparisons are not sufficient because the yields may vary during protein extraction. The authors should show quantification, e.g. by BCA, to ensure that loading is equal. In the manuscript the authors present tubulin as a loading control (for

example in figure S1B and other supplementary figures.) However, since X-ray film was used, there is no way to know that they are in the linear range of detection. Saturated exposure could mask loading differences. Therefore in figures S1B and S1E for example, there could be differences in Cut7 and Pkl1 expression that are not visible due to saturated exposure. There are no data included to show that the X-ray films that were scanned are in the linear range of detection. This is a problem common to X-ray based quantification approaches. The authors should address this.

We thank the reviewer for the comment. Indeed, the modest alterations in protein amounts in the studied mutants make protein concentration quantification highly recommended before gel loading. Therefore, we have slightly modified our protein extraction protocol, so that we could use a Bradford assay to quantify protein concentration in each sample. This ensured the equal loading of 5 µg of protein for each sample. In order to obtain quantifiable results, we have used a chemiluminescence imaging system (Fusion FX6 system, Vilber) for band signal detection.

1c. Related to the previous point, in figure S3 the tubulin bands appear very different between A, B, and C. 2 bands are visible in A, while only 1 is apparent in B and C. Can the authors explain the wildly different appearance of their loading controls?

We do not have an explanation for this effect. Although not frequent, in some cases we have found that our anti-tubulin antibody recognizes more than one band, which we have considered an artifact of the SDS-PAGE. Since we have been repeatedly using tubulin only as a loading control, and both bands had the same trend in terms of intensity, we decided to use them as a reference of protein amount in our samples. Nonetheless, in the new western blots shown in the paper and in the biological replicas used for the quantifications, a single tubulin band has been repeatedly detected.

2. Phos-tag gel analysis.

2a. In lines 199-203 in the discussion of the Phos-tag gel analysis of Cut7, the authors write "Our results showed an increased amount of slow migrating Cut7 species in mitotic blocked cells compared to

asynchronous cells, suggesting that Cut7 may be phosphorylated during mitosis. However, no differences in Cut7 migration were detected in ppe1Δ cells, both during the mitotic block or asynchronous growth (Figure S1C), reinforcing the idea that Cut7 may not be under direct control of PP6 phosphatase." This interpretation of the results seems over-simplified. A second band is visible in the low-temperature gel that appears to be absent in ppe1Δ. There also is a shift in of the 18 degree band relative to the 28 degree band in the phosphatase deletion. It's perplexing result that with removal of a phosphatase you lose bands. How do the authors explain this? The gel as presented also suggests there is a difference between the running size in the 28 degree condition because in the ppe1Δ is running lower. I suggest that this be discussed more extensively. For real comparison between the 28 deg bands of both strains should be run next to each other. Further, the percentage of the Phos-tag gels is not given. I suggest that the authors include this information. I also recommend that they run higher percentage gels to resolve these apparent differences.

We apologize if our explanation was not clear enough. Indeed, when we mentioned the "slower migrating Cut7 species", we meant this second band the reviewer mentions. If Cut7 were a target of Ppe1 dephosphorylation during mitosis, we would expect an accumulation of slower migrating bands in the *ppe1*Δ mutant, which is not the case. This supports our idea that Ppe1 does not directly control Cut7.

On the other hand, it is true that the blot shown in figure S1C, as presented, may lead to the conclusion that Cut7 may run lower in the 28ºC condition in the *ppe1*Δ mutant. We have repeated our PhosTag gel migrations in the sample order suggested by the reviewer and we could not repeatedly detect a difference in Cut7 migration at any condition as a result of the lack of Ppe1.

The information of PAA percentage in PhosTag gels is now included in the Materials and Methods section.

2b. In figure S1F why does Pkl1 look so different in the Phos-tag gel

from Cut7? It appears that the dumbbelling observed in the lanes would indicate that the lanes are over-loaded. And for the reasons described above, it's impossible to know if there is the same amount of protein in each lane because (1) there is no loading control shown and (2) apparent size differences could simply be caused or masked by loading differences.

We apologize for the presentation of figure S1F. PhosTag gel electrophoresis can be challenging and, even when running conditions remain the same, the outcome may vary from gel to gel. As previously mentioned, our protein extracts have now been measured by using a Bradford assay, to make sure that 5 µg of protein were loaded. We have added a loading control as suggested by the reviewer.

3. CLASP results. In lines 325-327, the authors state "Cls1 is an essential protein for spindle assembly ((56)), therefore we used a truncation of the PRC1 ortholog, ase1Δ(665-731), which lacks the interacting region with Cls1 and fails to recruit it properly to the mitotic spindle ((55))." The dependence of CLASP recruitment on Ase1 binding is controversial; contradictory results have shown that interaction with Ase1 is not required for Cls1 recruitment (Ebina et al Biology Open 2019). Other labs have also observed that Ase1 is not required for Cls1 recruitment in unpublished results. Therefore, the ase1Δ(665-731) strain not the best choice to perturb CLASP binding to spindle microtubules. The authors could consider a different strategy to perturb CLASP, but at least should discuss the interpretation of this result.

We strongly thank the reviewer for this comment. Indeed, the initial proposal that the mutant *ase1Δ(665-731)* had problems in Cls1 accumulation at the mitotic spindle (Bratman et al., Dev Cell, 2007) and that this *ase1* mutant suppressed *cut7-22* thermo-sensitivity drove us to the conclusion that reduced Cls1 amounts were behind this phenotype. Prompted by the reviewer comment, we tried to reduce Cls1 levels to see if this suppressed *cut7-22* phenotype. To do so,

we created a degron allele of Cls1, Cls1-3xAiD, based on the auxin-induced degron system (Zhang et al., G3, 2022).

The Cls1-3xAiD allele functions very efficiently, even at suboptimal concentrations of the auxin analog (10 nM), since cells harboring this allele die at 25ºC (Figure RB3). At 36ºC, however, *cls1-3xAiD* cells only die when auxin concentration is higher (100 nM). This suggests that, either the auxin-induced degron system is not completely effective at 36ºC, or that cells require less Cls1 in this condition. In any case, none of the conditions checked allowed the growth of any of the *cut7* thermo-sensitive alleles.

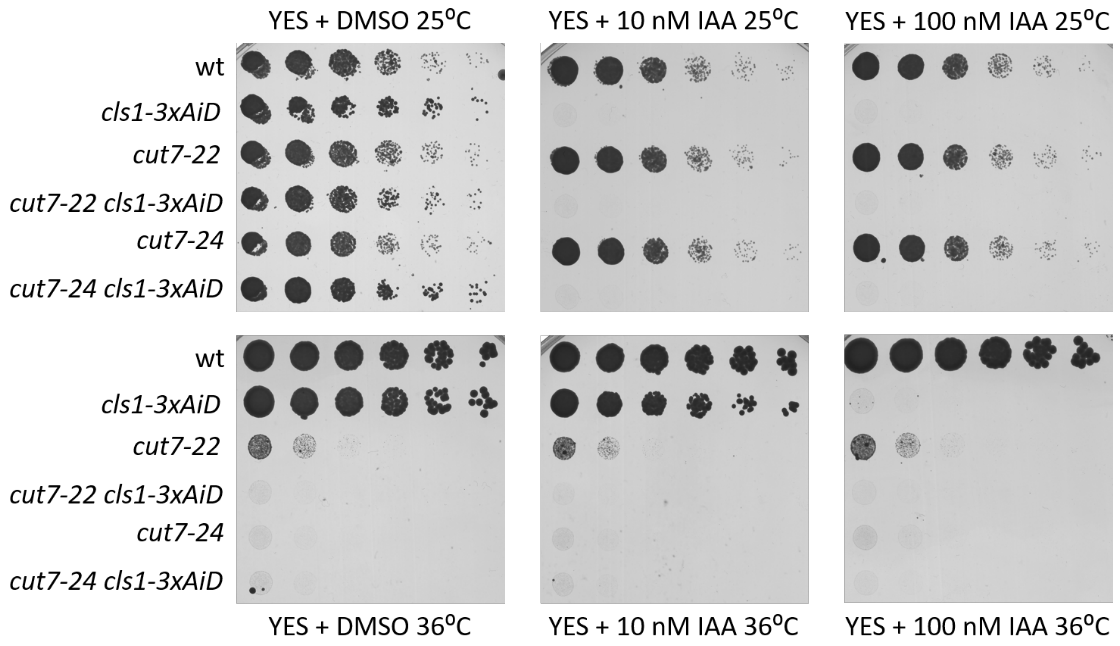

Figure RB3. Reduction in Cls1 levels does not suppress *cut7-22* or *cut7-24* thermo-sensitivity.

This result made us consider the possibility that the mutant *ase1Δ(665-731)* might not be a functional *ase1* allele, and that this might be the reason for the survival of *ase1Δ(665-731) cut7-22* cells at 36ºC. Indeed, we also found that full deletion of *ase1* suppresses *cut7-22* phenotype.

As a consequence, we checked if Ase1 levels were affected in the absence of PP6 or Elongator activity. Even though ase1 has a non-biased use of the codons coding for lysine (48% AAA vs. 52% AAG), it has a strong bias for the use of the codons coding for glutamate (76% GAA vs. 24% GAG), which also makes protein translation dependent on Elongator activity (Bauer et al., Cell Rep, 2012).

Altogether, these results made us take the decision of replacing the Cls1 data for that of Ase1, which is completely coherent with the context of the suppression of the *cut7-22* mutant thermo-sensitivity. Once again, we thank the reviewer for pinpointing a wrong interpretation of our initial results using the *ase1Δ(665-731)* mutant.

4. In lines 435-439 the authors refer to global phosphoproteomics analysis in the PP6 deletion. I did not find any data on these experiments in the paper. They should be presented.

We apologize for mentioning our attempt to find Ppe1 targets by using a global phospho-proteomics approach. These results are not presentable, since the huge differences in the global amount of proteins found in the *ppe1*Δ mutant compared to the wild type made the analysis impossible by the mass spectrometry platform of the CNB (Madrid). We have now omitted these lines.

Minor points:

5. The introduction presents a thorough and clear description of fission-yeast mitosis and the roles of Cut7, Pkl1, and other spindle proteins. However, there is only one paragraph that briefly discusses PP6 and Elongator, with the fission-yeast gene names for these proteins not mentioned. I believe that most readers of the paper will likely be more familiar with pombe mitotic proteins than with the roles of PP6 and Elongator in translation regulation, so more introduction to this process and proteins would be very helpful to the reader. I did not immediately recall, for example, the Elongator background presented in lines 222-233 and this made the paper more difficult to follow. Information on the importance of codon bias of AAA versus AAG for lysine as a potential contributor to perturbation of Elongator function is not presented until lines 299-300. I recommend that the authors discuss this more thoroughly in the introduction. A schematic of what's known about PP6 and Elongator early in the paper would also be helpful.

We acknowledge this comment of the reviewer. A paragraph at the end of the Introduction has been included to clarify the participation of Elongator in translation of AAA and AAG-enriched mRNAs, as well as the role of Ppe1 as a positive regulator of its activity (lines 132-137).

6. In the discussion in lines 424-434 the authors claim that the Phos-tag gels rule out a direct role of PP6 in Cut7 dephosphorylation seems to be to be an over-interpretation.

We have limited our conclusion to the lack of changes of Cut7 and Pkl1 dynamics in our microscopy experiments.

7. The cut7-24 mutation site discussed in the intro but the same is not done for cut7-22.

The *cut7-24* mutation site was presented in the Introduction, to explain the suppression screen. In the Introduction, the *cut7-22* is introduced to highlight the role of the kinesin 14 Klp2 in the force balance mechanism described by Yukawa and collaborators (G3, 2019). Nevertheless, we have added the following sentence in lines 123-124 to describe this *cut7* allele:

"Importantly, the ability of the *cut7* thermo-sensitive mutant *cut7-22* (which harbors the mutation P1021S at the C-terminal tail, close to phosphorylation sites."

8. I find it interesting that cut7-22 has more robust suppression of the ppe1 deletion compared to cut7-24; this result is even more dramatic in the elp3 deletion. What do the authors make of this? It would be interesting to discuss.

Both *cut7* alleles do not behave completely the same. In fact, *cut7-24* shows a more pronounced thermo-sensitivity as compared to *cut7-22*. This can also be observed in figure RB3. A possibility is that *cut7-24* compromises Cut7 activity, while *cut7-22* affects the phospho-regulation of this kinesin. However, there is no proof of this, and we find it too speculative to mention this in the text. Nevertheless, the differences in the extent of the rescue of *cut7* mutants by the lack of Ppe1 or by the absence of Elongator activity has already been discussed in lines 505-509, in which we argue that the stronger phenotypes observed in *ppe1*Δ as compared to *elp3*Δ may stem from the fact that Ppe1 may regulate other aspects of protein translation independently of the Elongator complex.

9. The order of presentation is confusing in several places. Figure 1E is discussed before B-D; why not move this panel earlier in the figure? In several places supplementary figures are discussed before main figures, for example S4 discussed before 4. In addition figure

S3F discussed before S3C and S3E. I suggest changing either the figure panel

or discussion order.

We agree that it was not too natural for the reading flow to mention before panel E to B in figure 1 or F to C in figure S3. Following the advice of Reviewer #1, some of the Supplementary Figure content has been moved to Main Figures (or vice versa), which had fixed the issue in figures 1 and 3. We do not find problematic to mention before the supplementary content of figure S4 than that of figure 4, so we have decided to keep it as it initially was.

10. The authors should check their use of the word astringent on line

501; perhaps they mean stringent?

This has been fixed.

11. In the supplement that caption of figure S2 is given after the

caption of figure S3.

We thank the reviewer for detecting this mistake, which has been fixed
